# The Lived Experiences of Students with Bullying in King Khalid University: A Qualitative Approach Study

**DOI:** 10.3390/ijerph21111445

**Published:** 2024-10-30

**Authors:** Yousef Zahrani

**Affiliations:** Department of Public Health, College of Applied Medical Sciences, Khamis Mushait, King Khalid University, Abha 61421, Saudi Arabia; y.zahrani@kku.edu.sa

**Keywords:** bullying, medical students, violence, educational environment, health system, anti-bullying, policies, Saudi Arabia

## Abstract

Psychological health and physiological health are crucial issues for educational institutes. However, bullying in universities remains a significant social issue that requires a deep understanding from the bully’s perspective. This qualitative study investigated students with lived experiences of bullying in two medical colleges at King Khalid University, Abha, Saudi Arabia. Nine male students participated in this study through semi-structured group discussions using open-ended questions. The discussions were transcribed, and the data were thematically analyzed. Four themes were identified: (i) the prevalence of bullying, (ii) bullying experiences, (iii) the institutional policy of bullying, and (iv) factors contributing to bullying. Findings showed that students lacked knowledge of their colleges’ anti-bullying policies, and verbal bullying was the prevalent form of bullying in interactions among students and between students and lecturers. In addition, cyberbullying was extended to digital platforms, as indicated by a number of participants. The participants also highlighted the significance of anti-bullying policies, believing that better awareness would empower students to address bullying more effectively. Recommendations were made for universities to implement anti-bullying policies and ensure their visibility to assist students in dealing with bullying situations.

## 1. Introduction

A supportive and safe educational environment is vital for students′ overall physical, emotional, and social well-being [1]. Despite efforts to promote a safe educational environment, bullying remains a critical issue in schools and universities, posing significant public health challenges worldwide. Each year, a number of students experience bullying behaviors, which negatively affects victims, perpetrators, and even bystanders [2,3]. According to Olweus, bullying is an intentional act of aggression meant to harm or intimidate others [4]. It often takes the form of repeated actions, encompassing verbal, non-verbal, physical, and emotional abuse [5]. This harmful behavior disrupts the learning environment, making it harder for students to engage in school activities and thrive academically [6].

### 1.1. This Study

This study explored bullying in King Khalid University (KKU), aiming to better understand how students perceive and experience bullying. Despite growing concerns about bullying in Saudi educational institutions, there is limited research on this topic. Additionally, the majority of existing studies rely on quantitative methods, which might overlook the personal and lived experiences of students. Hearing directly from students who are affected by bullying is essential for developing effective prevention and intervention strategies tailored to the Saudi university context.

### 1.2. Forms and Effects of Bullying

Bullying manifests in various forms, including verbal, physical, and social bullying, as well as cyberbullying [3]. Verbal bullying might involve name calling, body shaming, insults, or threats designed to belittle the victim [6]. Physical bullying includes acts of violence, such as pushing or hitting [7]. Social bullying, sometimes referred to as relational aggression, involves excluding someone from social groups or spreading rumors to tarnish someone’s reputation [3,8]. Cyberbullying, increasingly prevalent with the rise in digital platforms, involves using technology to harass or defame individuals, often anonymously, which can make it even more damaging [3,9,10]. In fact, the emotional toll of bullying is substantial [11].

The effects of bullying ripple beyond the individuals directly involved, affecting school communities and family systems [12]. Students who experience bullying often struggle to participate fully in school activities and, in some cases, may disengage entirely [13]. Research indicates that nearly half of students, across various educational stages, have been subjected to threats, harassment, or even violence [14,15].

Victims of bullying often experience depression, low self-esteem, and significant emotional stress, while bullies and witnesses are also impacted negatively [16]. Research has found that those who bully others are more likely to engage in antisocial behaviors and may be at a higher risk of substance abuse [16]. The growing severity of bullying incidents, particularly in the 1990s and early 2000s, prompted educators and policymakers to push for stricter anti-bullying measures [17,18]. In fact, if the anti-bullying policies and procedures for students are not revised at the university level, students will lack the necessary support and tools to recognize and report instances of bullying [18].

### 1.3. Bullying in Saudi Arabia and the Gulf Region

Bullying among university students in Saudi Arabia has been documented in various forms, including verbal bullying, physical bullying, and cyberbullying. A recent study conducted in Saudi Arabia to evaluate the quality of life among students reported that approximately 25% of students experience some form of bullying, with verbal bullying being the most prevalent [19]. This is consistent with global patterns, where verbal bullying is the most common type of bullying in higher education settings [20,21,22]. The study also found that cyberbullying, particularly through social media platforms, has emerged as a growing concern due to the increased use of digital technologies by university students [23].

The psychological consequences of bullying in academic environments can affect students’ academic performance, mental health, and overall well-being. Studies have shown that victims of bullying often experience heightened levels of anxiety, low self-esteem, depression, and feelings of isolation [3,24]. In addition, some studies from Saudi universities have revealed that students who face bullying report greater dissatisfaction with their academic experience than those who are not bullied [25]. Alrasheed et al. argued that the stigma associated with seeking mental health support in Saudi Arabia may worsen these outcomes, as students might hesitate to report incidents of bullying or pursue psychological assistance, increasing the risk of social isolation, which is particularly harmful in a collectivist culture where social integration is vital for individual identity and belonging [3,26,27].

In influential countries, cultural norms regarding masculinity result in an increased rate of physical bullying among male students at the university level [28]. This observation supports the notion that social hierarchies and traditional gender roles in Gulf societies significantly shape bullying behaviors.

A recent study in the United Arab Emirates (UAE) examined how the school environment influences bullying behaviors. The findings highlight the importance of fostering a positive school environment and implementing anti-bullying policies to mitigate bullying [29].

Despite growing awareness of bullying at the university level, responses to bullying within Saudi universities remain insufficient. Therefore, there is a need for more comprehensive and culturally appropriate interventions [23].

### 1.4. The Role of Universities in Addressing Bullying

Universities play a key role in preventing and addressing bullying, offering support not only to victims but also to those exhibiting bullying behaviors. Effective interventions often involve a combination of counseling, behavioral support, and institution-wide policies that promote a positive, inclusive learning environment [30]. Raising awareness of the impact of bullying is another critical responsibility for universities. Programs that promote empathy, kindness, and respect can foster a more compassionate school culture. Research suggests that the most successful anti-bullying initiatives involve the entire school community, including students, teachers, and parents, working together to reduce bullying incidents [31].

## 2. Materials and Methods

### 2.1. Study Design

This study used a qualitative research methodology, which was crucial for achieving the specified aims and objectives, particularly to explore the lived experiences of students involved directly or indirectly in bullying. Such an approach facilitates an in-depth understanding of phenomena and allows for the exploration of relevant aspects embedded within the educational context [32].

### 2.2. Setting, Participants, and Context

The research was conducted at KKU, in two medical colleges located in the southern region of Saudi Arabia. Participants were selected using a purposive sampling technique aimed at fulfilling the predefined selection criteria, specifically focusing on individuals with prior experiences of bullying. The inclusion criteria required that participants were currently enrolled at KKU and had completed a minimum of one academic year in any of the medical colleges. Nine male university students agreed to participate in this study. The participants were in their second to sixth year of study, and all identified as Saudi nationals and spoke Arabic.

### 2.3. Data Collection

Data were collected through group discussions involving the nine participants divided into three groups, each group comprising three individuals. The data were collected between April and September 2023, with each discussion lasting approximately 10 to 20 min. The discussions were structured by the researcher, and an interview protocol was established prior to their commencement. Participants were posed a series of pre-designed questions and allowed adequate time to provide thoughtful responses. By providing flexibility and convenience to the participants in responding, this approach afforded the researcher a comprehensive understanding of each participant’s experiences, thereby enhancing the quality and depth of the data gathered. First, the term “bullying” was defined, after which relevant questions were posed.

### 2.4. Recording Procedure

All group discussions were in Arabic and recorded using a digital audio-recording device. Prior to the interviews, participants were informed about the recording process, and they consented to the audio documentation of their voices. The discussions took place in a private office setting at KKU, ensuring confidentiality; participants were not required to disclose their names and were assured that participation was voluntary. The questions were specifically designed to focus on the participants′ experiences related to bullying, informed by a comprehensive literature review of the topic. In alignment with the best practices, participants received a briefing outlining the research objectives, which provided them with an opportunity to raise any questions or concerns prior to participation. Table 1 presents the group discussion protocol and associated questions.

### 2.5. Data Analysis

The data collected from the participants were transcribed and analyzed. The data were considered saturated when there was no new information. A thematic analysis of the students’ experiences with bullying was conducted in this study. To ensure a robust and methodologically sound approach to the thematic analysis, this study adhered to the six-step process proposed by Braun and Clarke [33], which provided a structure to the analysis of qualitative data, yet, at the same time, allowed for an in-depth and systematic exploration of material.

In the first step, for familiarization with the data and to develop a holistic understanding of their vastness and depths, the researcher went through the data repeatedly. Accordingly, this process started by making several passes through the raw data to deeply engage with them and then identify first-cut ideas and repeatable patterns. As Braun and Clarke emphasize, this stage is crucial in helping researchers develop an intuitive feel for their data prior to commencing more formal coding work, enabling them to immerse themselves in the richness and complexity of the participant accounts.

During the second step of preliminary coding, the data were read through several times, making a note to start spotting features that would be further analyzed. Coding is inductive because it allows for an a priori exploration of the content, while also being theory-driven, informed by the research questions. This process iteratively generates codes as the aim is to maintain a balance between accuracy and inclusivity, ensuring that important patterns are not overlooked.

The third step involved sorting through the codes to identify wider themes that summed up a pattern in the data. Themes are not descriptions of codes but, rather, a more interpretative level of the analysis to map out how the codes may relate to one another and what wider patterns are present across the data [34]. In this phase, early themes were identified by grouping related codes, and hence, a thematic map was developed.

The fourth step, reviewing themes, required an extensive rewrite of the original thematic framework to ensure consistency and clarity across the entire dataset. The researcher conducted this process at two levels: first, within each theme to ensure internal homogeneity and second, across themes to ensure external heterogeneity [35]. At this stage, the credibility of the themes was checked to ensure that they best represented the data and research questions in order to confirm that all relevant codes were nested within each thematic code.

In the fifth step, defining and naming all themes, themes were defined to clearly name them according to their essence. Braun and Clarke stress that simply using a label for codes is not sufficient and that the label should reflect the “essence”, further defining the theme; be clear; and be easily accessible to researchers [33]. Every theme was accurately defined to portray its salient components and then analyzed in relation to other themes so that the overall findings of the data could be adjusted.

The sixth step involved producing a report. The themes were linked into a findings section using appropriate quotes as exemplars for each theme.

All audio recordings of the participants’ group discussions were transcribed into Microsoft Word Version 2021. NVIVO software version 11 for Windows was used to generate codes, themes, and subthemes. 

#### 2.5.1. Rigor

The study ensured that the criteria of credibility, confirmability, reliability, and transferability in qualitative research were met in this study.

In this study, credibility and reliability were achieved through debriefing with a qualified bilingual peer to ensure the research process, analysis, findings, and data were consistent with the findings. Peer debriefing in qualitative research is used to review and assess transcripts, the emerging and final categories from those transcripts, and the final themes or findings of the study. In addition, after the data analysis, the participants were invited again and provided with a copy of the findings to confirm that their intended answers were accurately represented.

Confirmability refers to the objectivity of the study. The participants in this study were provided with an opportunity to speak freely, and the researcher listened without influencing their responses. This approach was used to ensure that the results were not affected by the researcher’s biases.

Transferability refers to the extent to which the findings of a study can be applied to similar contexts or settings. Transferability can be attained by data saturation. This may suggest that the results may be appropriate for similar situations in other contexts.

#### 2.5.2. Ethical Considerations

This research was approved by the KKU Bioethics Committee of Human Research (reference ECM#2022-2606). In addition, the study followed the framework of the Declaration of Helsinki, and all participants provided signed informed consent. Participating in this study was voluntary, and all participants were informed about the objectives of the study. All participants were also informed that their identities would be kept confidential.

## 3. Results

### 3.1. Demographic Data

The participants were nine current male students from the nursing and applied medical sciences colleges in the Khammis Mushait Campus, with 55.6% from nursing and 44.4% from applied medical sciences. The participants were in their third to sixth years of study, with those in the third year having the highest representation. Their ages ranged from 21 to 25 years. The nursing students were, on average, slightly older, with an average age of 23.4 years, compared to 23 years for applied medical sciences students. The mean age of all participants was 23.2 years (see Table 2).

### 3.2. Thematic Analysis

The analysis of bullying experiences among students at KKU revealed detailed insights into four major thematic areas: the prevalence of bullying, the impact of bullying, institutional bullying policies, and factors contributing to bullying. These findings shed light on the nuanced forms of bullying, its significant psychological and academic toll on students, and critical gaps in institutional responses:

1. Prevalence of bullying.
2. Bullying experiences.
3. Institutional policy of bullying.
4. Factors contributing to bullying.

#### 3.2.1. Prevalence of Bullying

The analysis indicated that verbal bullying is the most prevalent form encountered by students, with seven of nine participants identifying it as the primary type. The majority of participants reported observing bullying behaviors and positioning themselves as bystanders, with only two individuals sharing personal experiences. Verbal bullying typically involved body shaming, derogatory comments about academic performance, and mocking of language proficiency. Additionally, participant M6 highlighted the occurrence of cyberbullying, emphasizing that harassment often extends to digital platforms, particularly in WhatsApp groups.

Participant M4 shared a particularly emotional experience: “My friend had some hormonal changes; he looks soft, and other students started noticing that. They verbally bullied him. I also remember another student who wasn’t fluent in English. The lecturer began focusing on him, asking him questions, and sometimes calling him to the board to write his name. It was clearly intended to undermine him”.

Bullying is not limited to peer interactions; power imbalances between faculty and students also play a role. For instance, participant M7 recalled witnessing a lecturer mock a student with alopecia by giving him a nickname associated with a bald actor, which other students then adopted. Such incidents suggest that authority figures may inadvertently model bullying behaviors, normalizing it among students. In another case, participant N8 recounted how a lecturer ridiculed a student’s academic performance, stating, “The lecturer said, ‘You are dumb; how were you enrolled in the university?’” Such remarks not only undermine the student’s dignity but also foster a culture of public shaming. These accounts underscore the multifaceted nature of bullying experienced by students, involving both verbal and non-verbal elements.

#### 3.2.2. Bullying Experiences

The detrimental effects of bullying on students′ academic and psychological well-being were consistently emphasized across the responses. Participants frequently reported a direct correlation between bullying and their ability to focus on studies, attend classes, and engage in academic activities. Participant N3 described the isolating effect of bullying: “Bullied students become isolated; they come late to lectures, don’t participate, and are deliberately absent more”. This pattern of avoidance and disengagement was common among participants, leading to a marked decline in academic engagement.

For example, participant N2, who was bullied due to his weight, explained, “I try to avoid attending class. I come late, don’t participate, and sit in the back so no one can notice me”. Similarly, participant M7 shared how a once-active student began arriving late and wearing a cap to conceal his alopecia, further isolating himself. This cycle of withdrawal, driven by the fear of ridicule, often results in missed academic and social opportunities.

Some participants reported drastic measures in response to bullying, including withdrawing from academic commitments. Participant N1 mentioned, “A friend of mine dropped the course and later withdrew from the university because of bullying”.

The psychological effect of bullying is equally significant. Feelings of isolation, low self-esteem, and anxiety were common among respondents. Participant N9 shared a distressing experience: “Other students from the College of Medicine said, ‘Don’t waste your time. You’ll never be a doctor like us.’ This destroyed me. I underestimated myself, and I lost focus”. This narrative illustrates the profound emotional toll of bullying, often leading to self-doubt and a diminished sense of worth.

Moreover, long-term impacts on mental health and personal development were evident. Participant N9 described how persistent bullying by a lecturer eroded his confidence to the point where he began skipping classes and “lost a lot of marks”. Similarly, participant M4 recounted how a student consistently bullied for his English proficiency eventually withdrew from the university.

Participant M7 further described the behavioral changes in a student bullied for alopecia: “One student was bullied because of his hair. A lecturer compared him to a comedian TV actor. After this, the student covered his head every time he came to class, and if he couldn’t cover it, he skipped class”.

These narratives highlight the severe emotional and academic consequences of bullying, contributing to student disengagement and isolation. Moreover, these incidents illustrate how bullying undermines not only academic success but also the students’ capacity to persevere in higher education.

#### 3.2.3. Institutional Policy of Bullying

Despite the widespread nature of bullying, a clear lack of institutional support and awareness regarding how to report such incidents emerged from the findings. Many respondents expressed uncertainty or ignorance about the university’s policies on bullying, highlighting critical gaps in institutional communication. Participant N1, for instance, stated, “I’ve been at the KKU for more than three years and have never seen or read the university policy on bullying”. This sentiment was echoed by participant N2, who, after four years at the university, admitted, “I don’t know how to report bullying”.

This lack of clear guidance or access to reporting mechanisms exacerbates the issue, leaving students feeling unsupported. Participants M6 and N3 also revealed that they were unaware of formal procedures for reporting bullying, reflecting broader institutional failures in communicating available resources.

Even those familiar with anti-bullying policies found them vague and poorly communicated. Participant M4 recalled receiving an email vaguely referencing cyberbullying, but he felt it lacked specificity: “It was about cybersecurity, and I think bullying, but it failed to explain the consequences”. This lack of clarity reduces the perceived seriousness of the issue and fails to deter potential bullies.

Furthermore, the absence of a transparent reporting process creates a culture of silence around bullying. Several respondents, including participants M7 and N8, admitted that they did not know how to report bullying cases, even after multiple years at the university. This gap in the institutional framework suggests that students who experience or witness bullying have limited options for recourse, perpetuating the issue.

#### 3.2.4. Factors Contributing to Bullying

Participants offered several explanations for why bullying occurs, reflecting a range of social and psychological factors. A common theme was the need for individuals to assert dominance or superiority over others. Participant N1 suggested that bullying stems from a desire to “show off or display strength”. Similarly, participant M5 noted that students who feel “stronger or have a big group” may engage in bullying, underscoring the role of social power dynamics.

Some participants linked bullying to the perceived weakness or isolation of the victim. Participant N2 identified factors like a “weak personality” and “walking alone or social isolation” as making individuals more vulnerable. Participant N3 expanded on this by suggesting that bullying could be a “habit or lifestyle” linked to the bully’s psychological stress, implying that bullies may act out due to personal struggles.

Participant M4 pointed to social hierarchies, suggesting that students who believe that they are superior due to material possessions (e.g., “a good car”) or because of their prominent “family or tribe” may bully others to reinforce their perceived status. Participant M6 proposed that bullying may be cyclical, with bullies having been victims themselves: “Everyone who bullies has been bullied before”. Finally, participant N9 attributed bullying to “jealousy”, noting the role of envy in motivating such behavior.

These diverse perspectives offer a nuanced understanding of bullying, highlighting the complex interplay of social dominance, psychological factors, and personal experiences contributing to its prevalence.

## 4. Discussion

The findings of this study provide an important understanding of the prevalence and impact of bullying among students in two medical colleges at KKU. The discussion explores the findings according to the themes that were generated: the prevalence of bullying, bullying experiences, the institutional bullying policy, and factors contributing to bullying.

### 4.1. Bullying Prevalence

The results indicate that verbal bullying is the most common form of bullying in KKU, consistent with previous research conducted in Saudi universities and other global contexts [13,19,28,36].

In terms of prevalence, the majority of participants (88.9%) had either witnessed or directly experienced verbal bullying, which emphasizes the critical importance of the issue. This finding aligns with global studies, such as those by Al-Darmaki, Al Sabbah and Haroun [36], which suggest that bullying is not only prevalent but often impacts bystanders as much as direct victims. Verbal bullying is the most prevalent form and ranges from derogatory comments about academic performance to body shaming and mocking of language proficiency.

In addition to student-to-student bullying, the results indicate that some faculty members are reported as bullies as well. Bullying by lecturers includes negative comments about students’ academic performance and physical appearance. Such behaviors suggest that when the university academic staff engage in bullying, it can normalize the behavior, making it more difficult for students to report bullying incidents. Faculty bullying undermines the student–faculty relationship and fosters a culture of public shaming, which can further isolate students and reduce their engagement. This finding aligns with studies on workplace bullying in educational settings, where individuals in positions of power often model negative behaviors, perpetuating bullying across the institution [13].

Cyberbullying was less frequently reported in this study, acknowledged by only one participant. The rise in cyberbullying in educational settings has been documented globally, with research showing that digital platforms pose unique challenges for monitoring and intervention [37]. Universities must expand their bullying prevention strategies to encompass digital interactions, where harmful behaviors can occur unchecked.

### 4.2. Student Experiences of Bullying

The detrimental impact of bullying on students’ academic performance was a significant finding of this study. Participants reported that bullying affects academic performance in different forms. For example, victims often avoided classes, withdrew from academic activities, or reduced their engagement. These findings are consistent with prior research, such as that by Al-Buhairan et al., who found that bullying is a major factor contributing to lower academic performance in Saudi universities [38]. Rigby’s work further supports this by noting that bullying can lead to absenteeism and reduced motivation, as students attempt to avoid environments where bullying occurs [39].

This study also highlights the emotional toll bullying exacts on students, particularly verbal abuse related to language proficiency. Participants described a loss of confidence and increased anxiety, both of which hindered their ability to concentrate on their studies. This aligns with the findings of Kowalski et al., who documented similar patterns of diminished educational engagement and the prevalence of emotional distress among students subjected to bullying [40]. The psychological consequences of bullying, including anxiety, low self-esteem, and social withdrawal, should not be underestimated, as they directly influence students’ capacity to focus, participate, and thrive academically. Notably, verbal bullying related to language skills is especially harmful in academic settings, where linguistic proficiency is often tied to academic success.

To counteract these effects, universities need to provide more robust psychological and academic support services. Regular counseling sessions, peer support groups, and academic workshops focused on enhancing resilience and coping mechanisms could help students recover from the emotional and academic consequences of bullying. In addition, early intervention programs targeting students who exhibit signs of disengagement or absenteeism could prevent the long-term academic decline associated with bullying.

### 4.3. University Bullying Policy

One of the most worrying findings of this study is the widespread lack of awareness among students regarding the university’s anti-bullying policies. None of the participants were familiar with formal procedures for reporting bullying incidents. This finding is consistent with other studies that have highlighted a similar disconnect between policymakers and student awareness [28,41]. Indeed, this reflects a critical gap in university communication and raises questions about the efficacy of its efforts to protect students. Lack of awareness of anti-bullying policies can perpetuate a culture of silence around bullying. In fact, some researchers have reported that students are often reluctant to report bullying due to the fear of retaliation or skepticism about whether the university will take meaningful action [42]. This reluctance is exacerbated by the absence of clear reporting mechanisms, leaving students unsure about how to seek help. Universities need to ensure that anti-bullying policies are not only in place but also communicated effectively to the student body.

In addition to raising awareness, universities should focus on building trust in their reporting systems. Establishing clear, accessible channels for students to report bullying, coupled with guarantees of confidentiality, could increase reporting rates. Furthermore, faculty and staff must be trained to recognize bullying behaviors and respond promptly and appropriately. Visible action by the university when bullying is reported would demonstrate to the student body that such behavior is taken seriously, thereby increasing confidence in the institution’s ability to handle such cases.

### 4.4. Determinants of Bullying

This study identified several factors contributing to bullying, including social dominance, perceived superiority, and jealousy. These factors align with global trends, where bullying is often linked to social hierarchies and competition within academic environments. Participants noted that students from affluent backgrounds or those struggling academically are frequently targeted. This finding is consistent with previous studies that have demonstrated that social stratification in educational settings can exacerbate bullying [28].

The study also suggested that bullying may be cyclical in nature. Participants indicated that individuals who have been bullied in the past often engage in bullying behaviors themselves. This finding is in line with research by Bauman, Cross, and Walker, who found that individuals with a history of victimization are more likely to bully others as a means of regaining control or reasserting their power [43]. This cyclical nature of bullying underscores the complexity of the issue and the need for interventions that address the root causes of bullying behaviors, such as personal insecurities and psychological stress.

In addition to targeting bullies and victims, universities should consider implementing programs aimed at addressing the broader social environment in which bullying occurs. For example, by fostering a sense of community and support among students, peer-mentoring programs could help reduce the power imbalances that fuel bullying. Furthermore, workshops focused on empathy building and conflict resolution could help prevent bullying by teaching students how to manage interpersonal conflicts in a constructive and respectful manner.

## 5. Limitation

While this study provides valuable insights into bullying at KKU, several limitations need to be acknowledged. The first is the lack of representation of female students. Bullying experiences may vary between male and female students. This variation may be due to different gender roles, social dynamics, and cultural expectations. The second is the exclusion of academic staff, disregarding the perspectives of educational staff who may have witnessed incidents of bullying or been involved in investigating them. Research among faculty members may provide a broader understanding of bullying dynamics on campus, particularly when bullying occurs between students and staff. The third is language interpretation. The data were collected in Arabic and translated into English for the analysis. This may have led to translation bias, where subtle nuances or culturally specific meanings may have been missed or misinterpreted. Finally, there is the possibility of response bias. Participants may have provided socially desirable responses, especially given the cultural context in which the discussion of personal issues, such as abuse, may be seen as a weakness. This social desirability bias may have led to a reluctance on the part of the participants to fully disclose the extent of their experiences.

## 6. Conclusions

The findings of this study reflect the pervasiveness of bullying in universities in Saudi Arabia. Verbal bullying is an issue that significantly affects students’ emotional well-being and academic performance. Lack of knowledge of institutional policies among students and lack of reporting of bullying incidents further complicate this issue, highlighting the need for strong communication and support mechanisms in universities. Beyond the development of anti-bullying policies, institutions should ensure that these policies are widely communicated to and understood by students and faculty members.

Universities must establish clear guidelines for acceptable faculty behavior and provide lecturers with regular training in the professional and ethical treatment of students. Additionally, ensuring confidentiality and safety can encourage students to report faculty-related bullying, which can help address the power imbalance that often prevents students from speaking out against authority figures. Finally, further studies are required to include female students from different colleges and universities in Saudi Arabia.

## Figures and Tables

**Table 1:** Questions and protocols used in the group discussions.

Stage	Subject	Content and Questions
Introduction	Purpose	The aim of the study was explained, and bullying was defined.
Introduction	Ethical considerations	Participant agreement for recording the group discussions was obtained. The anonymity of participants was carefully maintained. All data were secured on a laptop with a password. The study was completely anonymous and voluntary.
Start	Group discussion questions	- How long have you been studying at KKU? - What is the prevalent form of bullying among students? - Tell me about your own experience with bullying. - What do you think are the reasons for bullying, and how can you face the different forms of bullying? - Tell me about the way you reported any bullying incident. - What are the effects of bullying among students? - Have you ever seen or read the university policy on bullying?
Closing	Final issue	Is there anything else you want to say or add?
	Acknowledgment	Thank you for your participation in this study.

**Table 2:** Participant demographics.

Participant	College	Year of Study	Age
N1	Nursing	3	21
N2	Nursing	4	24
N3	Nursing	4	24
M4	Applied medical sciences	5	23
M5	Applied medical sciences	3	22
M6	Applied medical sciences	6	24
M7	Applied medical sciences	3	23
N8	Nursing	2	23
N9	Nursing	3	25

## Data Availability

The original audio-recorded data used in this study are available from the corresponding author upon reasonable request.
